# Telomere Status of Advanced Non-Small-Cell Lung Cancer Offers a Novel Promising Prognostic and Predictive Biomarker

**DOI:** 10.3390/cancers15010290

**Published:** 2022-12-31

**Authors:** Eve Faugeras, Lauren Véronèse, Gaëlle Jeannin, Henri Janicot, Sébastien Bailly, Jacques-Olivier Bay, Bruno Pereira, Anne Cayre, Frédérique Penault-Llorca, Florent Cachin, Patrick Merle, Andrei Tchirkov

**Affiliations:** 1 Service de Pneumologie, CHU Clermont-Ferrand, 63000 Clermont-Ferrand, France; 2 UMR1240 “Imagerie Moléculaire et Stratégies Théranostiques”, Université Clermont Auvergne, INSERM, 63000 Clermont-Ferrand, France; 3 Service de Cytogénétique Médicale, CHU Clermont-Ferrand, 63000 Clermont-Ferrand, France; 4 EA7453 “Clonal Heterogeneity, Leukemic Environment, Therapy Resistance of Chronic Leukemias”, Université Clermont Auvergne, 63000 Clermont-Ferrand, France; 5 Service d’Hématologie Clinique, CHU Clermont-Ferrand, 63000 Clermont-Ferrand, France; 6 Délégation à la Recherche Clinique et à l’Innovation, CHU de Clermont-Ferrand, 63000 Clermont-Ferrand, France; 7 Centre Jean Perrin, Département de Pathologie, 63000 Clermont-Ferrand, France; 8 Service de Médecine Nucléaire, Centre Jean Perrin, 63000 Clermont-Ferrand, France

**Keywords:** advanced NSCLC, telomere length, *TERT*, shelterin complex, prognosis

## Abstract

**Simple Summary:**

Short, dysfunctional telomeres represent the genetic biomarkers of cancer. Studies in early-stage non-small-cell lung cancer (NSCLC) have shown that telomere length and telomerase levels are correlated with survival. In patients with advanced NSCLC, telomere status has not yet been investigated, and its clinical significance remains unknown. We studied telomere length and the expression of telomerase and shelterin genes in a cohort of 79 patients with advanced NSCLC, and evaluated these parameters as potential prognostic and predictive factors. Telomere shortening, high levels of telomerase and aberrant expression of shelterin genes *TRF2*, *RAP1* and *TIN2* were significantly correlated with shorter survival. Furthermore, a worse response to immunotherapy was observed in patients with shorter telomeres. The determination of telomere parameters in advanced NSCLC could be useful for individualized treatment decisions.

**Abstract:**

Telomere length appears to correlate with survival in early non-small-cell lung cancer (NSCLC), but the prognostic impact of telomere status in advanced NSCLC remains undetermined. Our purpose was to evaluate telomere parameters as prognostic and predictive biomarkers in advanced NSCLC. In 79 biopsies obtained before treatment, we analyzed the telomere length and expression of *TERT* and shelterin complex genes (*TRF1*, *TRF2*, *POT1*, *TPP1*, *RAP1*, and *TIN2*), using quantitative PCR. Non-responders to first-line chemotherapy were characterized by shorter telomeres and low *RAP1* expression (*p* = 0.0035 and *p* = 0.0069), and tended to show higher *TERT* levels (*p* = 0.058). In multivariate analysis, short telomeres were associated with reduced event-free (EFS, *p* = 0.0023) and overall survival (OS, *p* = 0.00041). *TERT* and *TRF2* overexpression correlated with poor EFS (*p* = 0.0069 and *p* = 0.00041) and OS (*p* = 0.0051 and *p* = 0.007). Low *RAP1* and *TIN2* expression-levels were linked to reduced EFS (*p* = 0.00032 and *p* = 0.0069) and OS (*p* = 0.000051 and *p* = 0.02). Short telomeres were also associated with decreased survival after nivolumab therapy (*p* = 0.097). Evaluation of telomere status in advanced NSCLC emerges as a useful biomarker that allows for the selection of patient groups with different clinical evolutions, to establish personalized treatment.

## 1. Introduction

Lung cancer is the leading cause of cancer-related deaths worldwide [1]. Non-small-cell lung cancer (NSCLC) is the most frequent lung cancer, accounting for 85% of all cases. NSCLC is frequently diagnosed at an advanced stage, and the overall prognosis is poor. Nevertheless, treatment responses and survival times are different in individual patients, which could be explained by biological heterogeneity of NSCLC [2]. This underlines the importance of biomarkers to identify subjects at higher risk of drug resistance and disease progression, who would benefit from more specific therapies. Furthermore, therapies targeting immune checkpoints have changed the treatment of NSCLC, enabling longer survival for patients with advanced disease. However, progression rates reported with nivolumab are above 30% [3,4], and approximately 14% of cases have hyperprogressive disease [5]. An early switch to salvage treatment in these patients should be considered, but no predictive factor has yet been identified.

Dysfunctional telomeres represent the genetic biomarkers of cancer [6,7]. Telomeres are nucleoprotein complexes essential for the protection of chromosomal ends. Due to incomplete DNA replication, telomeres shorten with every cell division [8]. Critical telomere shortening contributes to genomic instability and promotes cancerogenesis [9]. To maintain shortened telomeres, cancer cells activate telomerase, a specialized ribonucleoprotein that regenerates telomeric DNA, which enables survival and the limitless proliferation of tumors [10]. Upregulation of *TERT* (telomerase reverse transcriptase) gene leads to telomerase activation in cancers [11]. Telomeric DNA is protected by the shelterin protein complex, which regulates telomere length (TL) and prevents the inappropriate DNA damage response at chromosomal ends [12]. Shelterin contains six proteins: POT1 (protection of telomeres 1), TRF1 and TRF2 (telomeric repeat-binding factor 1 and 2), TIN2 (TRF1-interacting nuclear protein 2), RAP1 (repressor/activator protein 1) and TPP1 (TIN2 and POT1-interacting protein). Severe telomere shortening and abnormal expression of *TERT* and shelterin-complex genes were previously correlated with cancer prognosis [6,13,14].

Although relatively rare, telomere studies in NSCLC have shown that TL and telomerase levels were correlated with survival [15,16]. However, these clinical correlations have been evaluated only for early-stage surgically resected NSCLC. Moreover, only one study explored the prognostic value of shelterin gene expression in early-stage tumors [17]. In patients with advanced NSCLC, telomere status has not yet been investigated and its clinical significance remains unknown. In the present study, we measured TL and the expression of *TERT* and shelterin genes in a cohort of 79 patients with advanced NSCLC, and evaluated these parameters as potential prognostic and predictive factors.

## 2. Materials and Methods

### 2.1. Patients and Samples

This retrospective study was performed on 79 patients with histologically proven NSCLC from the Pulmonology Department of the University Hospital of Clermont-Ferrand. For this cohort, we collected the data on demographic characteristics, performance and smoking status, histological diagnosis, mutation status, TNM stage, treatment modalities, tumor-response evaluation, and follow-up. Tumor tissue samples were obtained using fibroscopy or transparietal biopsy and snap frozen until molecular analysis. Each sample was analyzed using histology, for diagnostic purposes, which also enabled us to verify the adequate tumor-cell content (>30%).

### 2.2. DNA and RNA Extraction

DNA and RNA were simultaneously extracted from snap-frozen tumor biopsies with the AllPrep DNA/RNA/miRNA Universal Kit (Qiagen, Courtaboeuf, France), in accordance with the manufacturer’s instructions.

### 2.3. Assessment of Telomere Length Using Quantitative PCR

Average telomere length in tumor DNA was evaluated with quantitative real-time PCR in a LightCycler 480 System (Roche Diagnostics, Meylan, France) using SYBR Green I technology (SYBR Green Kit, Roche Diagnostics), as described elsewhere [18]. This method measures the template amounts of telomere repeat (T) and a reference single-copy gene (S), which are then used to determine the relative telomere length as the T/S ratio [19]. Glyceraldehyde-3-phosphate dehydrogenase gene was used as the reference gene. Each PCR series also included normal DNA control. The T/S ratio of tumor samples was normalized relative to the ratio obtained in the control DNA. The normalized T/S ratio of 1 corresponds to the average length of telomeres which is identical to that of the normal control DNA, whereas ratios T/S < 1 indicate different degrees of telomeric shortening.

### 2.4. Quantitative RT-PCR for TERT and Shelterin Complex GENE expression

Total RNA was converted to complementary DNA by reverse transcription, using Superscript II reverse transcriptase (Invitrogen, Cergy-Pontoise, France), in accordance with the manufacturer’s instructions. The expression of *TERT* and the shelterin complex genes *TRF1*, *TRF2*, *POT1*, *TPP1*, *RAP1,* and *TIN2* were quantified using real-time RT-PCR in a LightCycler 480 System (Roche Diagnostics, Meylan, France), as described previously [18,20]. The normalized copy numbers (NCN) were expressed as the ratio of the numbers of transcript copies of the target and control (beta2-microglobulin) genes, multiplied by 100.

### 2.5. Statistical Analysis

Statistical analysis was performed using SEM software V1 [21]. The Student’s, Kruskal–Wallis, and Mann–Whitney tests were used for comparisons between the telomere data and clinical parameters. Event-free survival (EFS) was defined as the time between the date of diagnosis and the date of occurrence of the first event. The events were death from any cause, or clinical and/or radiological progression evaluated according to RECIST criteria [22]. Overall survival (OS) was defined as the time between the date of diagnosis and the date of death from any cause. Survival analysis was performed using the univariate Cox regression models, and survival curves were established in accordance with the Kaplan–Meier method and compared using the log-rank test. Multivariate Cox regression models were used to test the independent prognostic value of the telomere parameters. A sensitivity analysis was used to determine the best cutoff values associated with EFS and OS for the telomere parameters. To be precise, for each parameter value, EFS and OS were compared between cases that were below and above this value, using the Cox proportional hazards regression. The proportional hazard hypothesis was studied using Schoenfeld’s test. A sensitivity analysis was realized to find the best cutoff value for each parameter. An example of the best cutoff determination for TL is presented in Figure S1. An unsupervised hierarchical clustering was performed with SEM software [21]. Distances between clusters were calculated using 1-Pearson’s-correlation-coefficient values, and the dendrogram was constructed in accordance with to Ward’s algorithm.

## 3. Results

### 3.1. Patient Characteristics

The characteristics of the study cohort are presented in Table 1. Male smokers (active smokers or ex-smokers) represented the majority of the population. Adenocarcinoma was the most frequent type (58.2%) followed by squamous-cell carcinoma (31.7%). Eight patients had other histological types (six large-cell neuroendocrine carcinomas and two large-cell lung undifferentiated carcinomas). Two-thirds of NSCLC (70.8%) were metastatic at diagnosis. Thirteen patients had a KRAS-activating mutation, and four patients an EGFR mutation. Twenty-one patients with early NSCLC received primary local treatment with surgery (eleven patients), concomitant or sequential radiochemotherapy (eight patients) or radiotherapy alone (two patients). In advanced NSCLC, the first-line regimen was platinum-based chemotherapy (cisplatin or carboplatin) for fifty-two patients, and anti-EGFR tyrosine-kinase-inhibitor therapy for four patients with mutated EGFR.

### 3.2. Telomere Parameters in Early and Advanced NSCLC

We compared telomere characteristics at diagnosis between early (*n* = 21) and advanced NSCLC (*n* = 56) patients. TL was significantly shorter in advanced than in early NSCLC tumors (median T/S ratios: 0.4 vs. 0.59; *p* = 0.025). Expression levels of *RAP1* and *TPP1* were significantly lower in advanced NSCLC cases (median NCN: 11.65 vs. 30.74; *p* = 0.0020 and 33.7 vs. 106.3; *p* = 0.012, respectively). *TIN2* and *TRF1* tended to be more weakly expressed in advanced NSCLC cases (68.2 vs. 218.7; *p* = 0.054 and 49.5 vs. 177.6; *p* = 0.070, respectively). There was no significant difference in expression levels of *TERT*, *TRF2*, and *POT1* between early and advanced NSCLC cases.

### 3.3. Association of Telomere Parameters with the Response to First-Line Therapy in Advanced NSCLC

**Figure 1:**
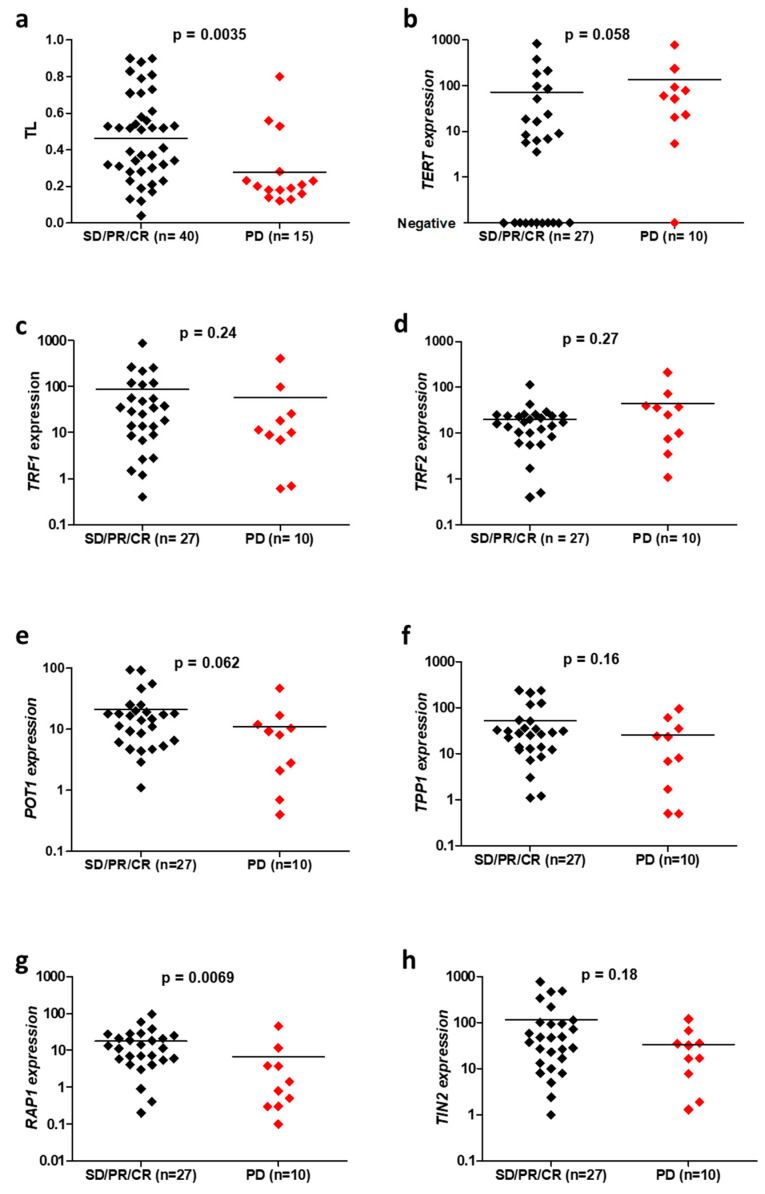
Comparison of telomeric parameters, including telomere length (TL) (**a**), *TERT* (**b**), *TRF1* (**c**), *TRF2* (**d**), *POT1* (**e**), *TPP1* (**f**), *RAP1* (**g**) and *TIN2* (**h**) expressions, depending on the response to the first-line chemotherapy for advanced NSCLC, in accordance with RECIST 1.1 criteria: progressive disease (PD) versus stable disease (SD), partial response (PR) or complete response (CR).

Fifty-six patients with advanced NSCLC were treated with first-line chemotherapy. A scanner assessment of response was performed after three months of treatment. TL was significantly shorter (*p* = 0.0035, Figure 1a) and *TERT* expression tended to be higher (*p* = 0.058, Figure 1b) in cases with progressive disease, in comparison to patients who responded to therapy or had stable disease. The expression of all shelterin complex genes except *TRF2* tended to be lower in the group of patients who progressed after first-line chemotherapy (Figure 1c–h). In particular, a very significant decrease was observed for RAP1 expression (*p* = 0.0069, Figure 1g).

### 3.4. Association of Telomere Parameters with Survival in Advanced NSCLC

For 56 patients who received first-line chemotherapy for advanced NSCLC, the associations of telomere parameters with EFS and OS were tested in univariate analyses, and the results are summarized in Table 2.

**Figure 2:**
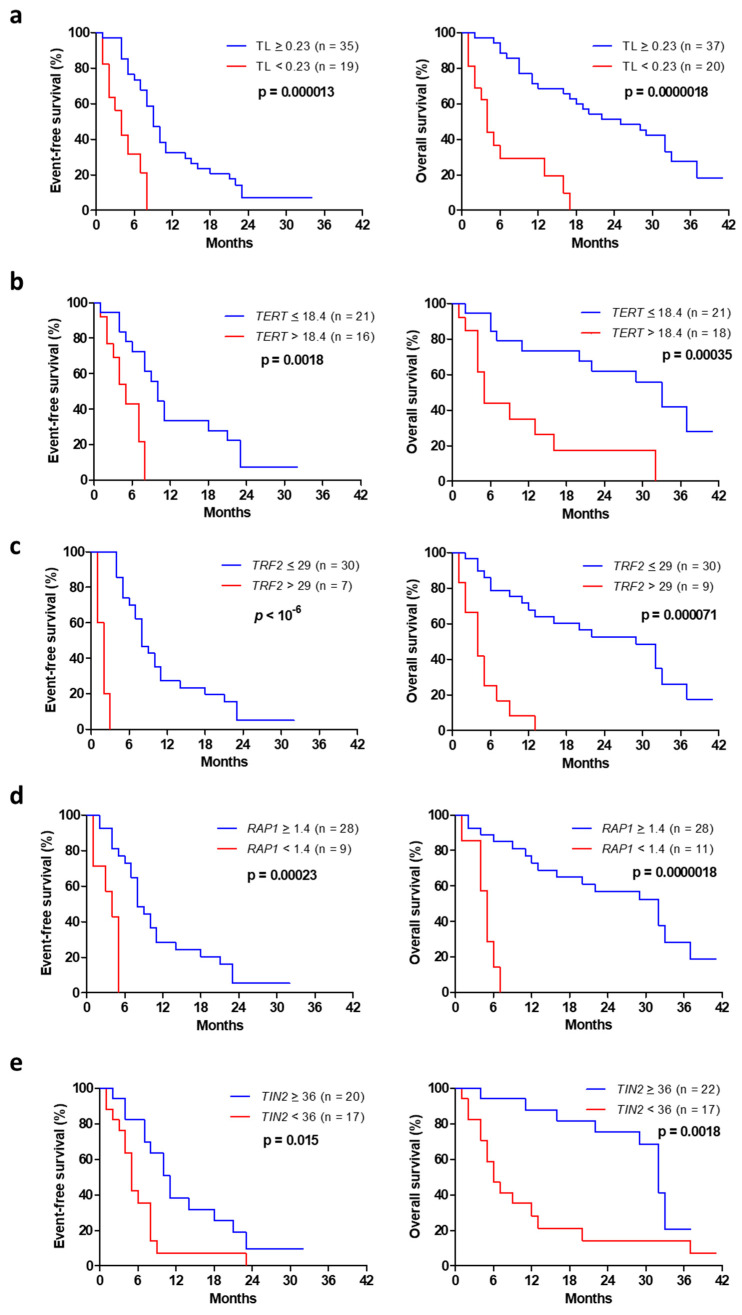
Event-free (left) and overall (right) survival from the date of diagnosis in advanced NSCLC as a function of telomere length (**a**), *TERT* expression (**b**), *TRF2* expression (**c**), *RAP1* expression (**d**) and *TIN2* expression (**e**).

Advanced NSCLC with short telomeres (<0.23) had significantly reduced EFS and OS, with a median EFS and OS of 4 months vs. 9 and 25 months, respectively, in the subgroup with longer telomeres (Figure 2a; *p* < 10^−3^). High *TERT* expression (>18.4) negatively affected EFS, with a median EFS and OS of 5 months compared with 10 and 32 months, respectively, in patients with low *TERT* expression (Figure 2b; *p* = 0.0018 and *p* = 0.00035). High tumor *TRF2* expression (>29) was significantly associated with reduced survival, with a median EFS and OS of 2 and 4 months vs. 8 and 29 months, respectively, in cases showing low *TRF2* expression (Figure 2c; *p* < 10^−6^ and *p* = 0.000071). Patients with low *RAP1* expression in their tumors (<1.4) showed a median EFS and OS of 4 months, whereas the median EFS and OS were 8 and 32 months, respectively, when *RAP1* expression was high (Figure 2d; *p* = 0.00023 and *p* = 0.0000018). Patients with low tumor *TIN2* expression (<36) also had a significantly worse clinical evolution (an EFS of 5 and OS of 6 months) compared with patients with high *TIN2* (Figure 2e; EFS of 11 and OS of 32 months; *p* = 0.015 and *p* = 0.0018).

A multivariate analysis, including age and performance status, indicated that short telomeres (<0.23), high *TERT* (>18.4), high *TRF2* (>29) and low levels of *RAP1* (<1.4) and *TIN2* (<36) expression remained significant prognostic factors (Table 3).

**Figure 3:**
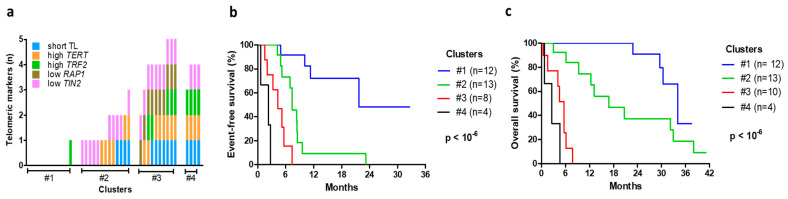
Telomeric markers combined together (**a**), statistically correlated with event-free (**b**) and overall survival (**c**).

In addition, an unsupervised hierarchical-clustering analysis was performed, to identify patient subgroups in accordance with the distribution of the telomeric markers (Figure S2 and Figure 3a). Survival analysis showed that the profiles of the telomeric biomarkers were highly correlated with EFS and OS (Figure 3b,c; *p* < 10^−6^). In particular, cluster #1 patients with negative telomeric markers had a longer EFS and OS (a median of 14 and 34 months). In cluster #2, the main features were low *TIN2* and high *TERT*, and patients had an intermediate prognosis (an EFS of 7.4 months and OS of 16.7 months). In clusters #3 and #4, tumors had multiple telomeric markers, and patient survival was considerably shortened (an EFS of 4.3 and 2.2 months and an OS of 5.5 and 2.5 months, respectively).

### 3.5. Correlation of Telomere Length with Survival in Advanced NSCLC Treated with Immunotherapy

**Figure 4:**
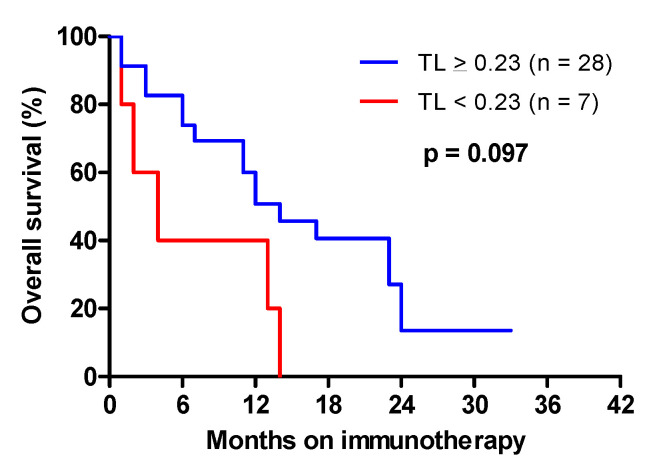
Overall survival from the date of the initiation of immunotherapy in advanced NSCLC as a function of telomere length.

In advanced NSCLC, nivolumab was given after the failure of at least one prior platinum-based chemotherapy regimen. Most patients received nivolumab in the second-line setting. To assess the impact of TL on the survival of patients treated with nivolumab, OS was defined as death from any cause, and determined from the date of the first infusion of nivolumab. In advanced NSCLC treated with nivolumab, short telomeres (<0.23) negatively affected overall survival, with a median OS of 4 months vs. 12 months when TL was ≥0.23 (Figure 4: HR = 2.33 [0.89–5.88], *p* = 0.097).

## 4. Discussion

Telomere attrition is one of the initiating events in lung cancerogenesis [23]. Subsequent activation of telomerase activity favors cancer-cell immortality and tumor progression. Short telomeres and the presence of telomerase activity in tumor tissue were significantly associated with reduced disease-free survival after curative surgery in early-stage NSCLC [15,16,24]. However, it is unknown whether telomere parameters may have prognostic value in advanced NSCLC. To address this issue, we assessed the impact of tumor telomere characteristics on treatment response and survival in advanced NSCLC patients, who represented the large majority of our cohort.

We found that telomeres were significantly shorter in advanced than in localized stages, which is in line with a previous report showing that telomeres were significantly shorter in the IIIB-IV stage than in the I-IIIA tumors [24]. We correlated telomere status with treatment response and survival for patients at advanced stages. At diagnosis, we identified approximately 35% of patients whose tumors displayed very short telomeres. In this sub-group, the response to first-line chemotherapy was considerably worse, and EFS, as well as OS, was significantly shorter than in patients with longer telomeres in their tumors. Thus, enhanced telomere shortening was strongly associated with tumor resistance and a worse prognosis in advanced NSCLC.

Among lung-cancer cases, 85% of tumors express telomerase independently of the disease stage [16]. We did not observe any significant difference in *TERT* expression levels between early and advanced cases. In early, resectable NSCLC, the presence of telomerase activity was shown to negatively affect survival after surgery [24,25]. In advanced NSCLC, we found that the EFS and OS of patients with higher *TERT* levels were significantly worse. Also, higher *TERT* expression was detected in patients who were resistant to first-line chemotherapy. Thus, telomerase overexpression is strongly associated with tumor aggressiveness in advanced NSCLC.

Expression levels of shelterin complex genes were previously studied in lung cancer, but only *TRF1*, *TRF2*, *POT1*, and *RAP1* mRNA were measured in early NSCLC, and no consistent pattern of expression was identified [17,24,26]. We examined the expression of all six core members of shelterin in early and advanced disease, and found significantly lower expression of *RAP1* and *TPP1* and reduced expression of *TIN2* and *TRF1* in advanced NSCLC. No change was found for *TRF2* and *POT1* expression. Interestingly, *RAP1* expression was significantly lower in patients who did not respond to chemotherapy. Moreover, we found that expression levels of *TRF2*, *RAP1*, and *TIN2* were significantly correlated with the survival of patients with advanced NSCLC.

High *TRF2* expression was significantly associated with shorter PFS and OS. TRF2 has been increasingly recognized as involved in telomere maintenance and DNA damage response (DDR) [27]. Short telomeres are perceived as double-strand DNA breaks and induce DDR mediated by p53/ATM (ataxia-telangiectasia mutated) proteins. TRF2 has been shown to inhibit the action of the ATM protein [28,29]. Increased expression of *TRF2* in response to short telomeres is a factor of poor prognosis, because ATM-dependent apoptosis may be more strongly inhibited, and this allows the cell to survive and cancer to progress [30]. High expression of *TRF2* is associated with poor prognosis in other cancers such as hepatocellular carcinomas or advanced-stage cervical cancers [31,32].

Low expression of *RAP1* at diagnosis was a poor-prognostic factor, with reduced EFS and OS. RAP1 protects the telomere ends from the attacks by the DNA repair mechanisms similarly to other proteins of the shelterin complex, but by different mechanisms [33]. In particular, RAP1 plays an important role in the repression of aberrant homology-directed DNA repair at the telomeres, which prevents tumor cells from further telomere loss and genomic rearrangements [34]. Altered *RAP1* expression has been found in multiple types of human cancer, including NSCLC [35,36,37,38]. In early NSCLC, low *RAP1* expression has been previously correlated with short survival [17]. Our observation shows that the decreased *RAP1* expression is correlated with the resistance to first-line chemotherapy and poor survival, in advanced NSCLC.

We found that low *TIN2* expression was associated with reduced EFS and OS. To our knowledge, this is the first study showing the prognostic impact of low *TIN2* in NSCLC. TIN2 binds to TRF1, TRF2, and TPP1, thus forming a bridge between the double-stranded telomeric DNA-related proteins and those related to single-stranded sequences [12]. The TRF1-TIN2 interaction is mediated by the TRFH domain and a pattern specific for the C-terminal region of TIN2, while the N-terminal region of TIN2 associates with the hinge domain of TRF2 [39]. When these interactions are simultaneous, TIN2 connects TRF1 to TRF2, and this link contributes to the stabilization of TRF2 on telomeres. TIN2 also recruits TPP1 (and thus POT1) via a third interaction site located in its N-terminal region [40]. TIN2 is a key component of the shelterin complex, and depletion of TIN2 has a powerful destabilizing effect [41]. *TIN2* deficiency in aging mice was shown to lead to telomere fragility, accumulation of DNA damage at chromosomal ends and an enhanced lymphoma formation [42]. This mechanism may be relevant to the development of human malignancies. As compared to normal tissue, *TIN2* transcription levels are lower in tumor tissue in breast cancer [43], gastric cancer [44] and chronic lymphocytic leukemia [20]. Low *TIN2* expression was also shown to be a factor of poor prognosis in chronic lymphocytic leukemia [45,46,47].

Immune checkpoint inhibitors have changed the management of treatment of advanced NSCLC, with improved survival and better tolerance compared with standard chemotherapy. Nivolumab is a humanized monoclonal antibody against programmed death 1 (PD-1), approved as second-line treatment in patients with advanced NSCLC [48]. Current tests to predict the response to immunotherapy such as tumor PD-L1 overexpression or mutation burden have so far yielded inconsistent results [49]. We found that in patients treated with nivolumab, short telomeres negatively affected survival. To our knowledge, this is the first evidence that telomere length could be a prognostic factor and, possibly, a response predictor in advanced NSCLC treated with immunotherapy. Recently, genomic instability involving arm and whole-chromosome copy-number aberration has been proposed as a predictive biomarker for cancer immunotherapy [50]. Telomere dysfunctions can lead to the formation of cancer with a heavily rearranged genome [9]. Indeed, the unprotected chromosome ends generate end-to-end fusions and dicentric chromosomes, leading to many forms of genome instability, including global chromosomal copy-number alterations [51]. Therefore, the presence of short telomeres is consistent with enhanced genome instability of this particular type, which could lead to a poor response to nivolumab in advanced NSCLC.

Patients with newly diagnosed advanced NSCLC are currently tested for the presence of actionable mutations in tumor DNA [52]. In patients with no identified driver, RNA-based testing is considered, to search for druggable fusion genes. Including the telomeric biomarkers into the clinical-routine setting would be relatively easy, since the tests can be performed on very small amounts of already available DNA and RNA samples. The analytical performance of the tests has to be certified in accordance with medical laboratory quality-requirements. The tests can be recommended for all patients, to provide prognostic and predictive information. In addition, telomere targeting has been proposed for prolonging disease control of therapy-resistant NSCLC patients, and clinical benefits from telomerase inhibition seem to be greater in patients with tumors showing a more pronounced telomere dysfunction [53,54].

## 5. Conclusions

We have shown that short telomere-length, high levels of *TERT* and *TRF2*, and low expression of *RAP1* and *TIN2* are significantly associated with poor EFS and OS in advanced NSCLC. Moreover, enhanced telomere shortening in tumors could be a biomarker for worse survival in patients treated with nivolumab. Thus, evaluation of telomere status emerges as a useful molecular tool that allows for the selection of groups of NSCLC patients with different clinical evolutions, to establish personalized therapy protocols.

## Figures and Tables

**Table 1:** Demographic and disease characteristics of the NSCLC cohort (*n* = 79).

Characteristics	Median (Range) or No. (%)
Median age, years	65 (43–82)
Men	38 (48.1)
Smoking	74 (93.7
Adenocarcinoma	46 (58.2)
Squamous cell carcinoma	25 (31.7)
Other types	8 (10.1)
Stages	
I and II	11 (13.9)
III with local treatment	10 (12.7)
III with chemotherapy	1 (1.3)
IV	56 (70.8)
Not known ^1^	1 (1.3)
Genetics	
*KRAS* mutation	13 (16.5)
*EGFR* mutation	4 (5.1)
*ALK* translocation	1 (1.3)
Treatments	
None ^1^	2 (2.5)
1st line treatment	
Local	
Surgery	11 (13.9)
Radiotherapy alone	2 (2.5)
Radiochemotherapy	8 (10.1)
Chemotherapy	52 (65.8)
Tyrosine kinase inhibitors	4 (5.1)
2nd line nivolumab	36 (45.6)
3rd line nivolumab	2 (2.5)
4th line nivolumab	1 (1.3)

^1^ Early death.

**Table 2:** Univariate analyses for event-free and overall survival in the advanced NSCLC cohort.

Variable	Univariate Cox for EFS		Univariate Cox for OS	
	HR (95% CI)	*p*-Value	HR (95% CI)	*p*-Value
Telomere length				
Decreased (<0.34; median)	1.85 (1.00–3.33)	0.049	1.92 (1.03–3.57)	0.04
Short (<0.23; best cutoff)	6.67 (2.86–14.29)	0.0000062	6.25 (3.03–14.29)	0.0000017
TERT expression				
Increased (>12.8; median)	1.95 (0.88–4.34)	0.1	3.01 (1.24–7.03)	0.011
High (>18.4; best cutoff)	4.84 (1.81–12.93)	0.0016	4.75 (2.02–11.21)	0.00037
Shelterin gene expression				
Decreased *TRF1* (<18.2; median)	1.39 (0.68–2.86)	0.37	1.72 (0.77–3.85)	0.18
Increased *TRF2* (>20.4; median)	1.11 (0.53–2.32)	0.78	1.28 (0.57–2.21)	0.55
High *TRF2* (>29; best cutoff)	9.40 (3.43–25.76)	0.000013	8.68 (2.77–27.25)	0.00021
Decreased *POT1* (<11; median)	1.25 (0.60–2.56)	0.56	1.54 (0.68–3.45)	0.3
Decreased *TPP1* (<24.5; median)	2.13 (0.051)	0.051	1.43 (0.61–3.23)	0.4
Decreased *RAP1* (<7; median)	2.70 (1.22–6.25)	0.015	1.61 (0.73–3.57)	0.035
Low *RAP1* (<1.4; best cutoff)	8.33 (2.70–25.00)	0.00022	14.29 (4.00–50.00)	0.016
Decreased *TIN2* (<31.9; median)	2.63 (1.22–5.56)	0.013	2.04 (0.87–4.76)	0.016
Low *TIN2* (<36; best cutoff)	2.94 (1.35–6.25)	0.0065	3.85 (1.61–9.09)	0.0026

EFS: event-free survival; OS: overall survival; HR: hazard ratio, 95% CI: 95% confidence interval.

**Table 3:** Multivariate analyses for EFS and OS in the advanced NSCLC cohort.

Variable	Multivariate Cox for EFS *		Multivariate Cox for OS *	
	HR (95% CI)	*p*-Value	HR (95% CI)	*p*-Value
Short telomeres	4.35 (1.69–11.11)	0.0023	5.26 (2.08–12.5)	0.00041
High *TERT*	4.12 (1.47–11.53)	0.0069	3.99 (1.51–10.50)	0.0051
High *TRF2*	7.31 (2.43–22.01)	0.00041	8.24 (2.43–27.93)	0.007
Low *RAP1*	9.09 (2.70–33.33)	0.00032	14.29 (3.85–50.00)	0.000051
Low *TIN2*	3.33 (1.39–8.33)	0.0069	3.03 (1.19–7.69)	0.02

* Adjusted for age and performance status.

## Data Availability

The data supporting reported results are available from the corresponding author on request.

## Supplementary Materials

The following supporting information can be downloaded at: https://www.mdpi.com/article/10.3390/cancers15010290/s1, Figure S1: The best cutoff determination for telomere length values. Forest-plot for hazard ratios (HR) and 95% confidence intervals (95% CI) associated with the overall survival (OS) for each value of telomere length (between 0.17 and 0.39). This sensitivity analysis shows that the best cutoff of TL associated with the OS is 0.23. Figure S2: An unsupervised hierarchical clustering was performed by combining telomere length (TL) and TERT, TRF2, RAP1 and TIN2 gene expression as dichotomic parameters relative to best cutoff values used for survival analysis. Negative prognostic factors (short TL, high TERT and TRF2, low RAP1 and TIN2) are shown in red and their absence is indicated in green. The different color intensities of dichotomic parameters reflect normalized values and not original binary (0/1) values.
