# Supplementary material for: Telomere Status of Advanced Non-Small-Cell Lung Cancer Offers a Novel Promising Prognostic and Predictive Biomarker
Source: Cancers (Basel). 2022 Dec 31;15(1):290. doi: 10.3390/cancers15010290 (PMC9818321; doi:10.3390/cancers15010290)
Supplement: Supplementary file 1 [file cancers-15-00290-s001.zip › Figure S1.pdf]

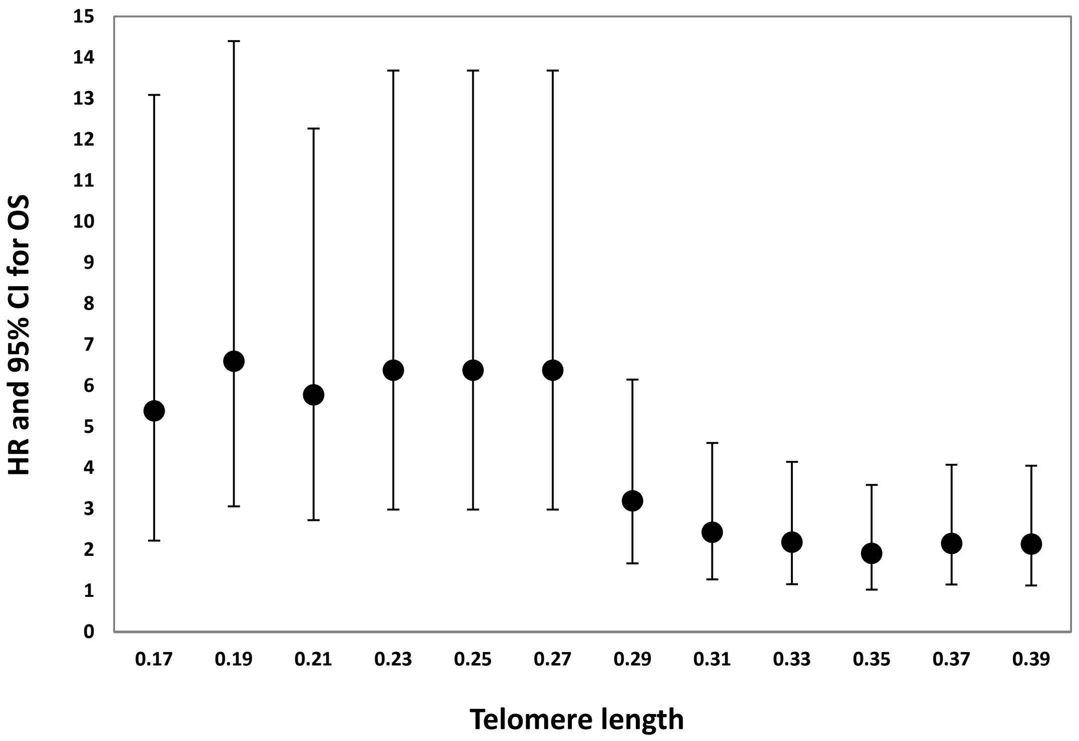

Figure S1

The best cutoff determination for telomere length values. Forest-plot for hazard ratios (HR) and 95% confidence intervals (95% CI) associated to the overall survival (OS) for each value of telomere length (between 0.17 and 0.39). This sensitivity analysis shows that the best cutoff of TL associated to the OS is 0.23. The « plateau effect » exists between 0.23 and 0.27, because no patient showed 0.25 or 0.27 values, and these thresholds define the same group
