# Supplementary material for: Telomere Status of Advanced Non-Small-Cell Lung Cancer Offers a Novel Promising Prognostic and Predictive Biomarker
Source: Cancers (Basel). 2022 Dec 31;15(1):290. doi: 10.3390/cancers15010290 (PMC9818321; doi:10.3390/cancers15010290)
Supplement: Supplementary file 1 [file cancers-15-00290-s001.zip › Figure S2.pdf]

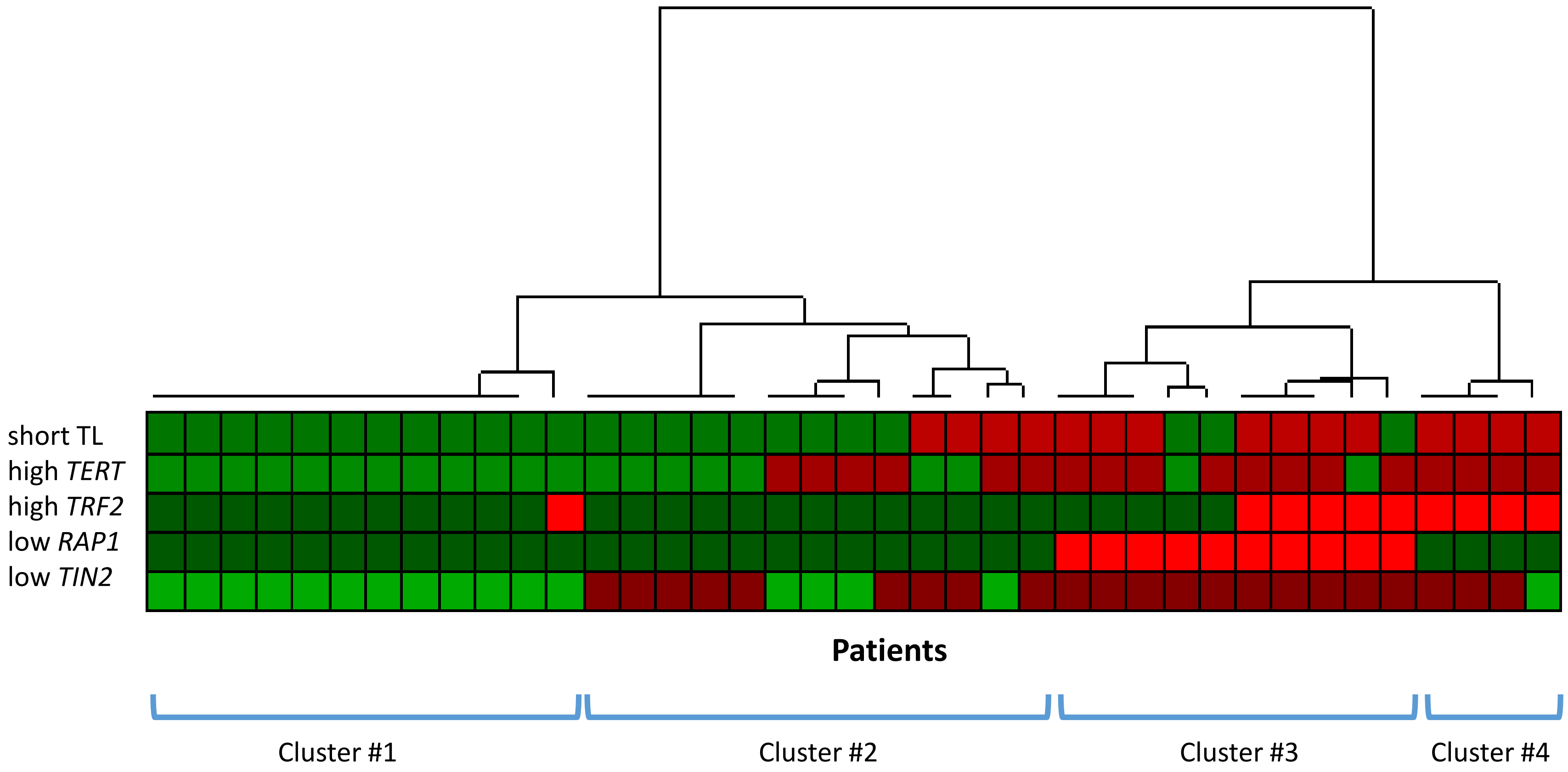

Figure S2

An unsupervised hierarchical clustering was performed by combining telomere length (TL) and *TERT*, *TRF2, RAP1* and *TIN2* gene expression as dichotomic parameters relative to best cutoff values used for survival analysis. Negative prognostic factors (short TL, high *TERT* and *TRF2*, low *RAP1* and *TIN2*) are shown in red and their absence is indicated in green. The different color intensities of dichotomic parameters reflect normalized values and not original binary (0/1) values.
